# Mini-DAQ: A lightweight, low-cost, high resolution, data acquisition system for wave energy converter testing

**DOI:** 10.1016/j.ohx.2022.e00332

**Published:** 2022-06-21

**Authors:** Bret Bosma, Ryan Coe, Giorgio Bacelli, Ted Brekken, Budi Gunawan

**Affiliations:** a O.H. Hinsdale Wave Research Laboratory, Oregon State University, Corvallis, OR; b Sandia National Laboratories, Albuquerque, NM; c Electrical & Computer Engineering, Oregon State University, Corvallis, OR

**Keywords:** System monitoring, Data logging, Open hardware, EtherCAT, Renewable energy, Energy, Electrical engineering, Wave energy

## Abstract

As part of the development process, scaled testing of wave energy converter devices are necessary to prove a concept, study hydrodynamics, and validate control system approaches. Creating a low-cost, small, lightweight data acquisition system suitable for scaled testing is often a barrier for wave energy converter developers’ ability to test such devices. This paper outlines an open-source solution to these issues, which can be customized based on specific needs. This will help developers with limited resources along a path toward commercialization.

## Graphical abstract

**Figure:**
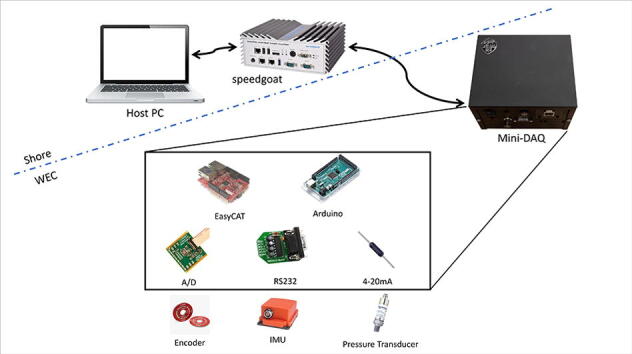

## Specifications table

**Table:** 

Hardware name	Open-source low-cost light-weight data acquisition system
Subject area	• Engineering and materials science Educational tools and open source alternatives to existing infrastructure
Hardware type	• Measuring physical properties and in-lab sensors Field measurements and sensors Electrical engineering and computer science
Closest commercial analog	• Data acquisition and control systems
Open source license	• GNU General Public License (GPL)
Cost of hardware	$250
Source file repository	https://doi.org/10.5281/zenodo.6012684

## Hardware in context

Ocean wave energy presents a largely untapped renewable energy resource with tremendous potential. Although the concept of generating electricity from ocean waves is not new, its path to commercialization is ongoing and has faced many challenges. One such challenge has been in the small-scale prototyping necessary to validating and advancing device concepts. Relevant challenges in scaled power take-off (PTO) testing are outlined in [1]. Many commercially available control systems suitable for small scale prototyping are heavy, have a large footprint, and are cost prohibitive. The alternative of locating the data acquisition system in a remote location and connecting by cables is not favorable, as the dynamics of a small model-scale wave energy converter (WEC) can easily by affected by cable connections and presents an increased opportunity for noise due to electromagnetic fields (EMF). Often, they are a barrier for researchers and small developers eager to prove their concept.

We present an open-source light-weight data acquisition system suitable for small scale WEC systems. The system outlined here highlights a few commonly used signals, but the framework allows for expansion depending on specific needs. WEC concepts vary widely, and sensor requirements are almost never standardized between devices. Having a flexible framework where sensors and actuators can be added and removed easily is a technical requirement of the project.

Bringing signal conditioning off the device can save in space and weight on the device but can also create an unwieldy umbilical and have signal degradation and noise issues. Thus, signal conditioning should occur onboard as close to the sensor and actuator locations. The proposed miniature data acquisition system or Mini-DAQ meets these requirements. The backbone of the Mini-DAQ system is the EtherCAT[2] communications protocol which provides deterministic messaging and allows the transmission of data over an ethernet cable to shore. All data is transported on one cable, significantly reducing the umbilical compared to systems where the signal conditioning is on the shore. Diameter, weight, and bend radius concerns are improved when transferring data on a single cable. Power requirements also need to be met, but reduction of the data to a single cable is highly beneficial. Furthermore, the deterministic messaging properties of EtherCAT are of great importance to real-time control and is a common application of such systems[3], [4]. In fact, the MiniDAQ framework provides the basis for expanding to a real-time control system.

EtherCAT has emerged as a preferred communication platform for industrial communications[5], [6]. It has been widely adopted, with devices such as many motor drives having EtherCAT capabilities built in. Where appropriate, there are many commercial EtherCAT components available that could fit a user’s need, so long as they meet space and budget requirements.

This study aims to provide an open-source easily replicable data acquisition system suitable for small scale WEC research. A set of core features are presented. Additional customization will be required, depending on the application. For example power supply, enclosure, and mounting specifications will need to be specified on a case by case situation.

## Hardware description

The Mini-DAQ is an open-source compact data acquisition system intended for laboratory testing of wave energy converters. It interfaces to a variety of sensors to collect data to be sent on an EtherCAT bus to a control and data logging computer. The design is intended to be relatively simple to assemble and easily customizable and replicable. It allows for a lightweight inexpensive compact package to be on the WEC device, close to the sensors and actuators and is highly customizable.

The Mini-DAQ design has interface capability and code for three commonly used sensors in WEC systems.

• Four channel differential ± 10 V 16-bit analog to digital (A/D) converter input
• RS232 serial communication (for example, for interface with an inertial measurement unit (IMU))
• Implementation of 4–20 mA pressure sensor capture using precision resistor

Additional sensors that have been tested but are outside the scope of this project.

• Eight-channel ± 10 V 16-bit digital to analog (D/A) converter output
• Wheatstone bridge low level voltage input
• Synchronous Serial Interface (SSI)
• Digital I/O
• Quadrature encoder input
• Two channel 4–20 mA 16-bit current measurement for measuring pressure sensors for example

Additional information regarding additional testing of sensors within this framework is available in [7].

In this implementation, an Arduino Mega 2560[8] is used to interface with signal conditioning. The EasyCAT shield for Arduino[9] allows the Arduino to put data onto the EtherCAT network for logging and control. This constitutes a secondary node on the EtherCAT network. The primary EtherCAT node used in this example is executed by a Speedgoat computer running compiled MATLAB/Simulink code. Potential future work includes the development of a low-cost primary EtherCAT node to be paired with the MiniDAQ.

## Design files summary

**Table:** 

Design file name	File type	Open source license	Location of the file
Arduino/Arduino.ino	Arduino Firmware	GPL 3.0	https://doi.org/10.5281/zenodo.6012684
Simulink/MiniDAQ.slx	Simulink Model	GPL 3.0	https://doi.org/10.5281/zenodo.6012684
Simulink/initMiniDAQ.m	MATLAB m-file	GPL 3.0	https://doi.org/10.5281/zenodo.6012684
Simulink/MiniDAQapp.mlapp	MATLAB app	GPL 3.0	https://doi.org/10.5281/zenodo.6012684
Simulink/postProcess.m	MATLAB m-file	GPL 3.0	https://doi.org/10.5281/zenodo.6012684
TwinCAT/MiniDAQ1kHzDC.xml	EtherCAT xml	GPL 3.0	https://doi.org/10.5281/zenodo.6012684

• **Arduino/Arduino.ino:** Arduino sketch acquiring data and transferring to the EtherCAT network.
• **Simulink/MiniDAQ.slx:** Simulink file running on the Speedgoat as the primary EtherCAT node displaying and recording data for post-processing.
• **Simulink/initMiniDAQ.m:** MATLAB initialization script for configuring and starting the Speedgoat.
• **Simulink/MiniDAQapp.mlapp:** MATLAB app user interface used to start and stop simulation and visualize data.
• **Simulink/postProcess.m:** MATLAB post processing script to pull data from the Speedgoat, and organize and save data for post-processing.
• **TwinCAT/MiniDAQ1kHzDC.xml:** EtherCAT xml file needed by Simulink to define the network.

## Bill of materials summary

**Table:** 

Designator	Component	Number	Cost per unit -currency	Total cost - currency	Source of materials	Material type
Controller board	Arduino Mega 2560	1	$40.30	$40.30	https://store-usa.arduino.cc/products/arduino-mega-2560-rev3?selectedStore = us	Electrical Component
EtherCAT board	EasyCAT	1	$55.59	$55.59	https://www.bausano.net/shop/en/home/1-arduino-ethercat.html	Electrical Component
ADC Evaluation Board	A/D converter	1	$55.00	$55.00	https://www.mouser.com/ProductDetail/Analog-Devices/DC682A?qs = ytflclh7QUU1sdPvjEFblQ%3D%3D	Electrical Component
Serial converter	RS232 converter	1	$7.90	$7.90	https://www.digikey.com/en/products/detail/mikroelektronika/MIKROE-222/4495513?s = N4IgTCBcDaILYEMAeYDMEC6BfIA	Electrical Component
Precision resistor	Metal Film Resistor	1	$4.39	$4.39	https://www.digikey.com/en/products/detail/vishay-dale/PTF65250R00AXEK/6604108?s = N4IgTCBcDaIAoBUBiA2ArGNAGASlrAggBoCiA0iALoC%2BQA	Electrical Component

## Build instructions

**Fig. 1:**
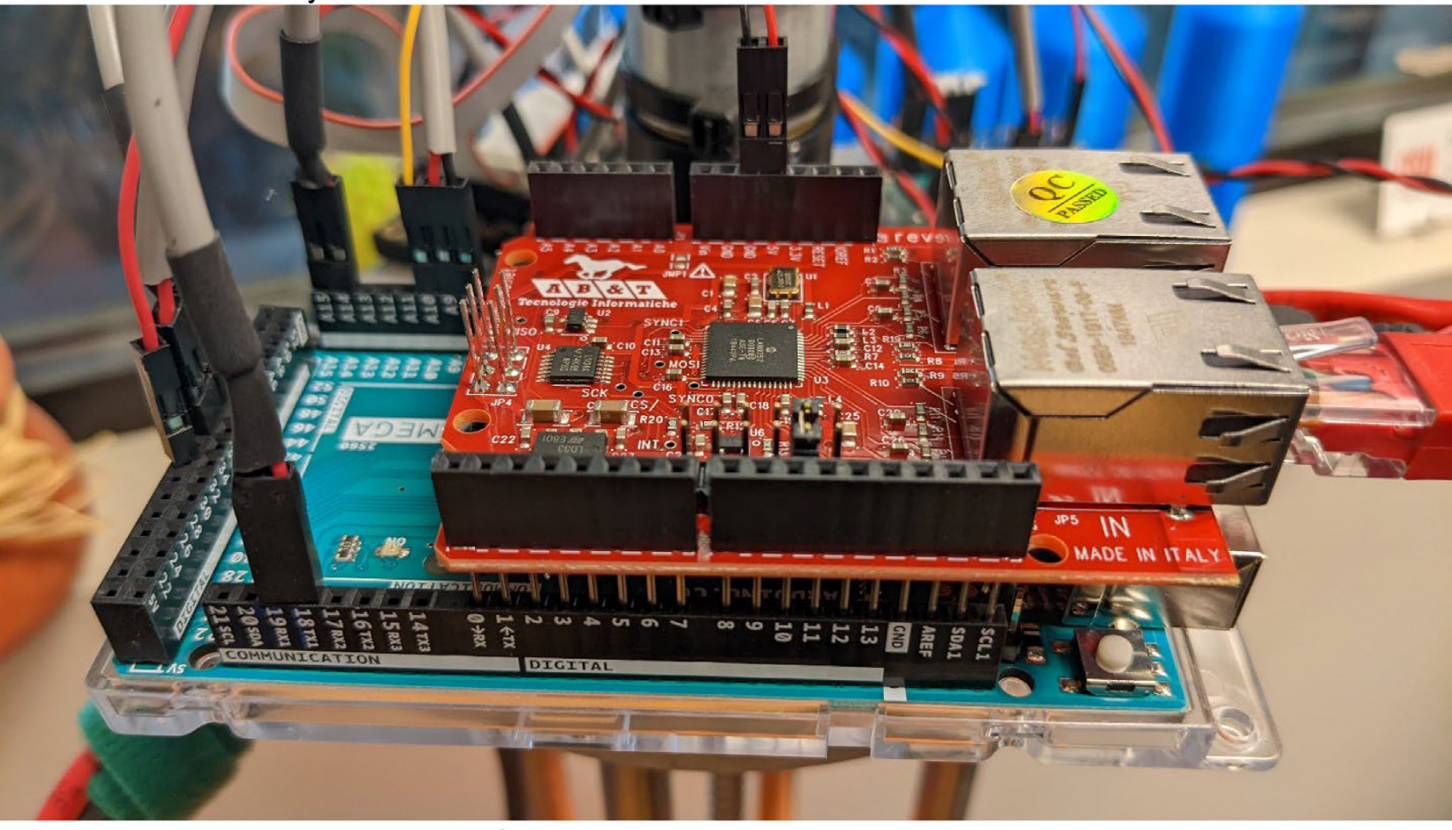
EasyCAT board installed on Arduino Mega 2560.
• Connect the ADC evaluation board to controller board as shown in Fig. 2 with pin assignments in Table 1. This can be done by modifying the included ribbon cable, or by removing the connector from the ADC evaluation board and soldering jumper wires directly to the board. Verify that JP1 on the ADC evaluation board is set to EXT, and JP2 is set to INT.Table 2

• Install the EasyCAT board onto the controller board as shown in Fig. 1.

**Fig. 2:**
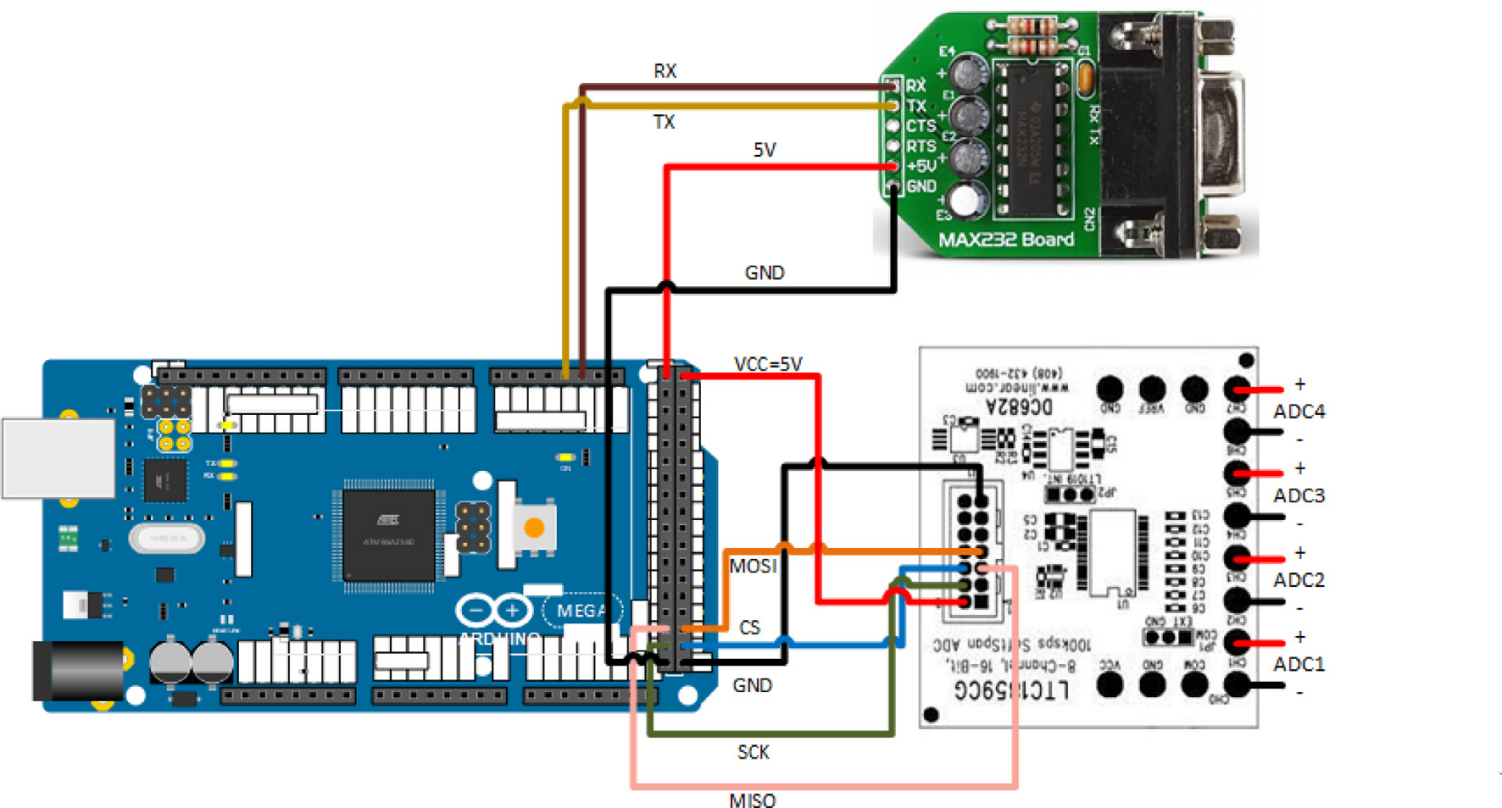
Wiring of ADC evaluation board and serial converter board to controller board.

**Table 1:** 

Arduino	Max232	DC682
18	TX	–
19	RX	–
5 V	+5V	–
GND	GND	–
5 V	–	2
52	–	4
50	–	5
53	–	6
51	–	7
GND	–	13

**Table 2:** 

	MiniDAQ	HWRL	scope
Mean (V)	2.7112	2.7178	2.7190
% error	0.2606	0.0168	0.0271
Standard deviation	0.0005	0.0002	0.0488
• Connect MAX232 Board to the controller board as shown in Fig. 2 and Table 1 using jumper wires.
• Connect the Arduinio Mega 2560 via USB to a computer and program with code Arduino/Arduino.ino.
• Remove USB cable and connect power with 7–12 V power supply via the barrel connector.
• Connect xsens IMU to the MAX232 Board via 9 pin D-Sub connector.
• Connect 4–20 mA pressure sensor to precision resistor as shown in Fig. 3, and connect resistor to ADC1 input from Fig. 2.

**Fig. 3:**
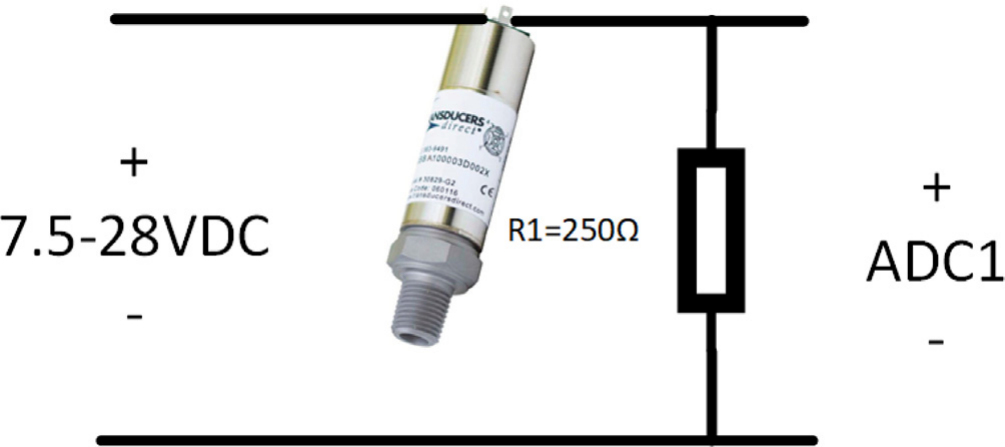
Circuit for connecting 4–20 mA sensor to analog input.

## Operation instructions

• Configure and connect the primary EtherCAT node (in this example PC host machine and Speedgoat).
• Ensure that the Simulink initialization block in MiniDAQ.slx points to the file MiniDAQ1kHzDC.xml.
• Connect the Speedgoat to the MiniDAQ via an Ethernet cable making sure to plug into the “IN” port on the EasyCAT board.
• Customize and run ‘Simulink/initMiniDAQ.m’ from MATLAB

## Validation and characterization

As a test of the quality of measurement the Mini-DAQ can provide, a direct comparison with an industry standard data acquisition system and oscilloscope was undertaken. The analog to digital converter was used for this study. Four channels of input data were fed from their source to the three acquisition systems. The signals fed are a voltage standard, random delay square wave, sine wave, and bandlimited white noise.

All tests were performed at the O.H. Hinsdale Wave Research Laboratory (HWRL) at Oregon State University. The data acquisition system used in this facility includes a National Instruments PXI system which includes a chassis and controller. Analog data acquisition is controlled by a NI PXI-6259 M−series 16-bit multifunction DAQ module. This module is then connected to a SCXI-1143 Butterworth anti-aliasing filter module. This module is then fronted with an SCXI-1305 terminal block that take ±5V differential inputs from analog channels via 50 Ω coaxial cable with BNC connectors. The SCXI-1143 Butterworth anti-aliasing filters are set with cutoff frequency at ¼ the sampling rate. Unless specifically noted, a sampling rate of 10 ms was used for data capture. Heretofore this data acquisition will be referred to as HWRL.

The oscilloscope used for additional comparison of results is the Tektronix DPO7054 Digital Phosphor Oscilloscope. Captured data was saved locally to the hard drive of the oscilloscope and transferred via USB flash drive for post processing. The data was captured at a sample rate of 10 ms. Heretofore this oscilloscope will be referred to as scope.

**Fig. 4:**
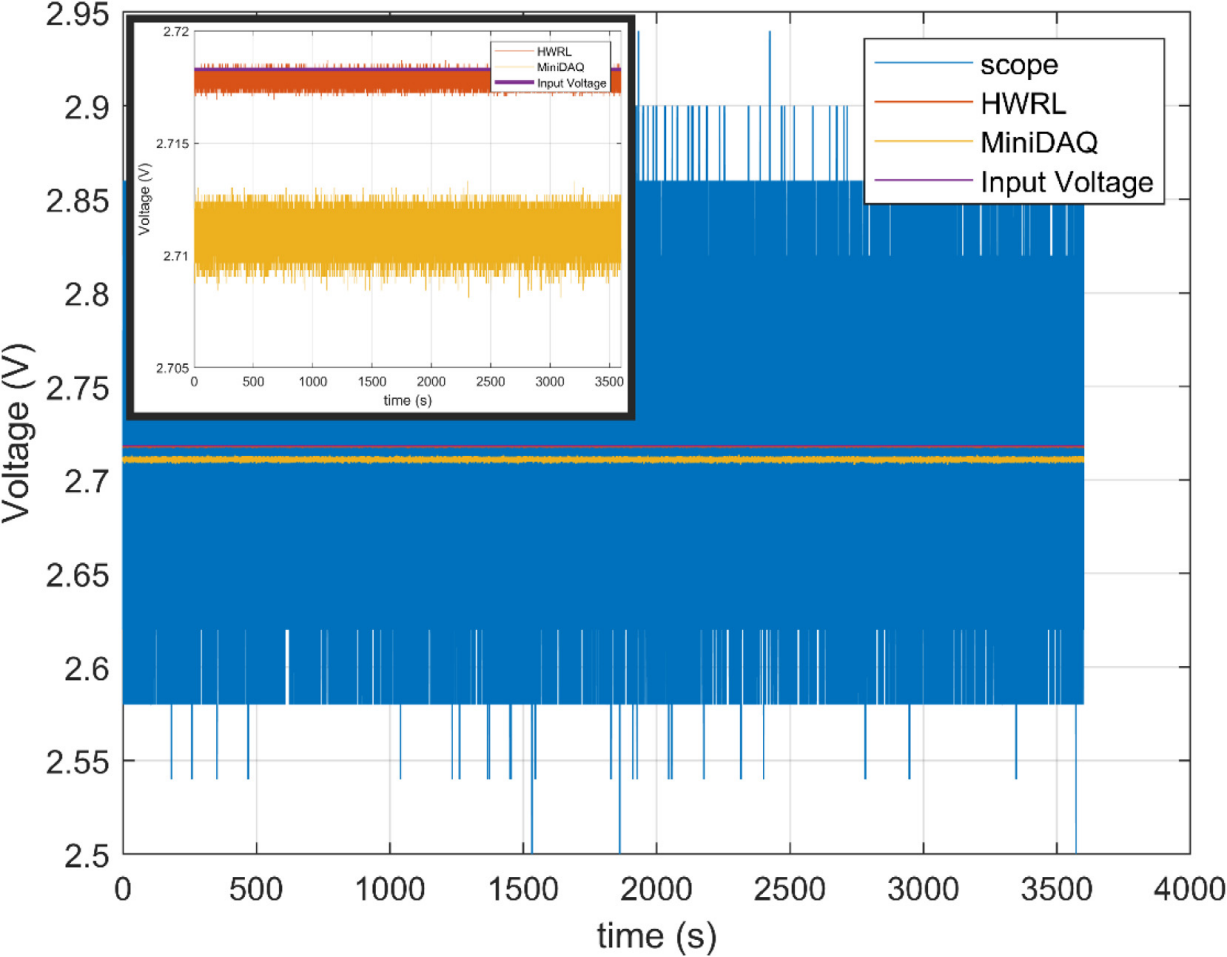
Recording of voltage standard on the scope, HWRL DAQ, and Mini-DAQ. Prescribed input voltage shown for reference. Inlay removes scope plot to show relationship more clearly between the MiniDAQ and HWRL signals.

The voltage standard is a piece of test equipment typically used in calibration and validation of data acquisition systems and provides a stable constant prescribed voltage. For this test an Analogic AN3100 with a current NIST traceable calibration was used. The voltage was set to a value of 2.71828 V and recorded for one hour with results shown in Fig. 4. Statistical analysis was performed on the three recorded signals of interest and results are shown in Table 1. While the Mini-DAQ has a higher percent error and standard deviation compared to the HWRL and scope, the result indicates values acceptable for most applications.

**Fig. 5:**
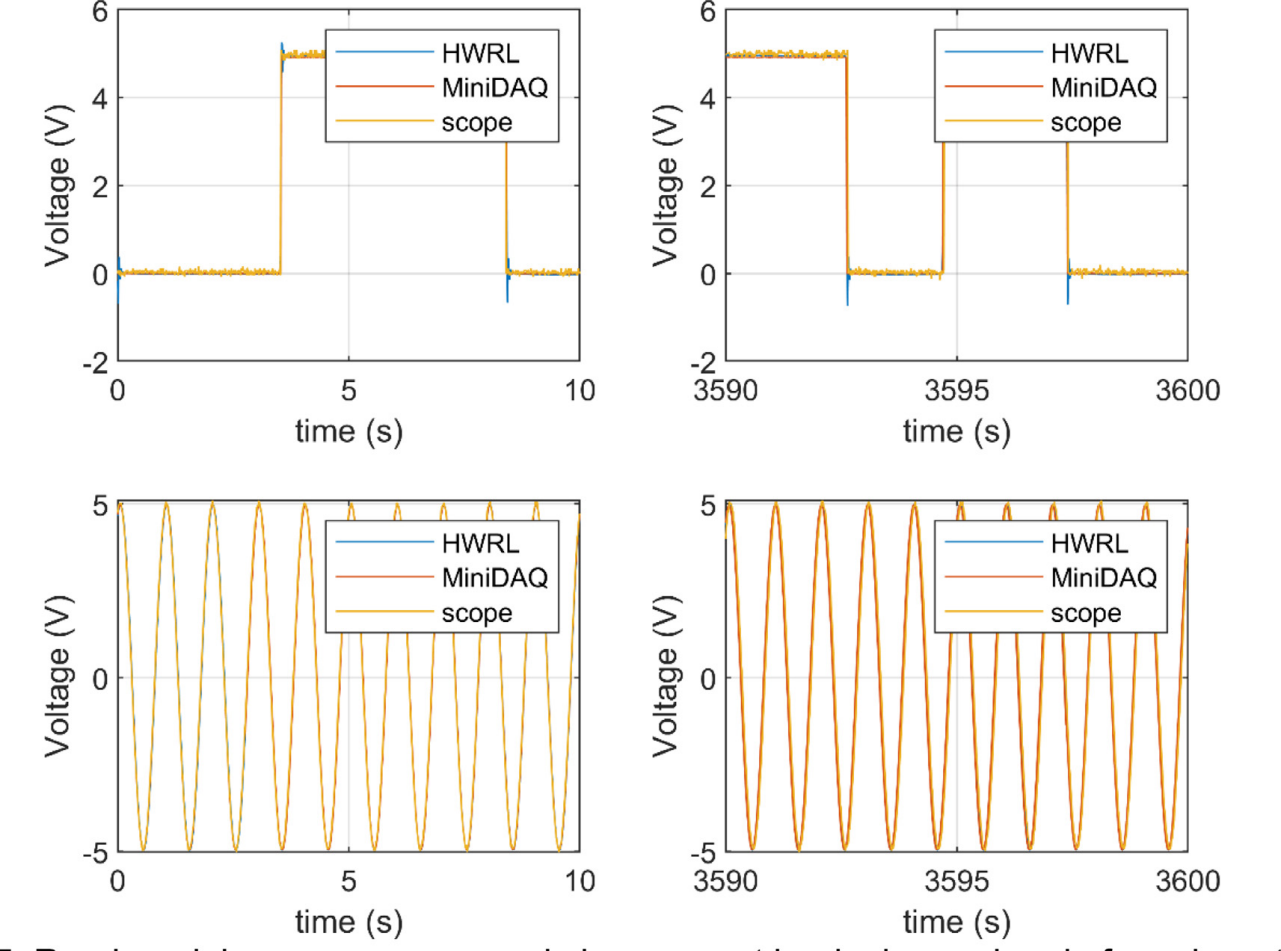
Random delay square wave and sine wave at beginning and end of one hour trial. Notice minimal phase shift after an hour of running.

The next signal of interest recorded was a random delay square wave. This is a signal used in the lab to synchronize datasets captured from different sources. The output signal is split and captured by all relevant systems which allows for time alignment in post processing. In this case, the first rising edge was used to align the three different data captures. The upper left of Fig. 5 shows the beginning of the record with signals aligned on the first rising edge. The upper right of Fig. 5 shows the end of the hour-long record with minimal phase shift between the signals. The lower left of Fig. 5 shows the sine wave at the start of the record and lower right the end, again with minimal phase shift.

**Fig. 6:**
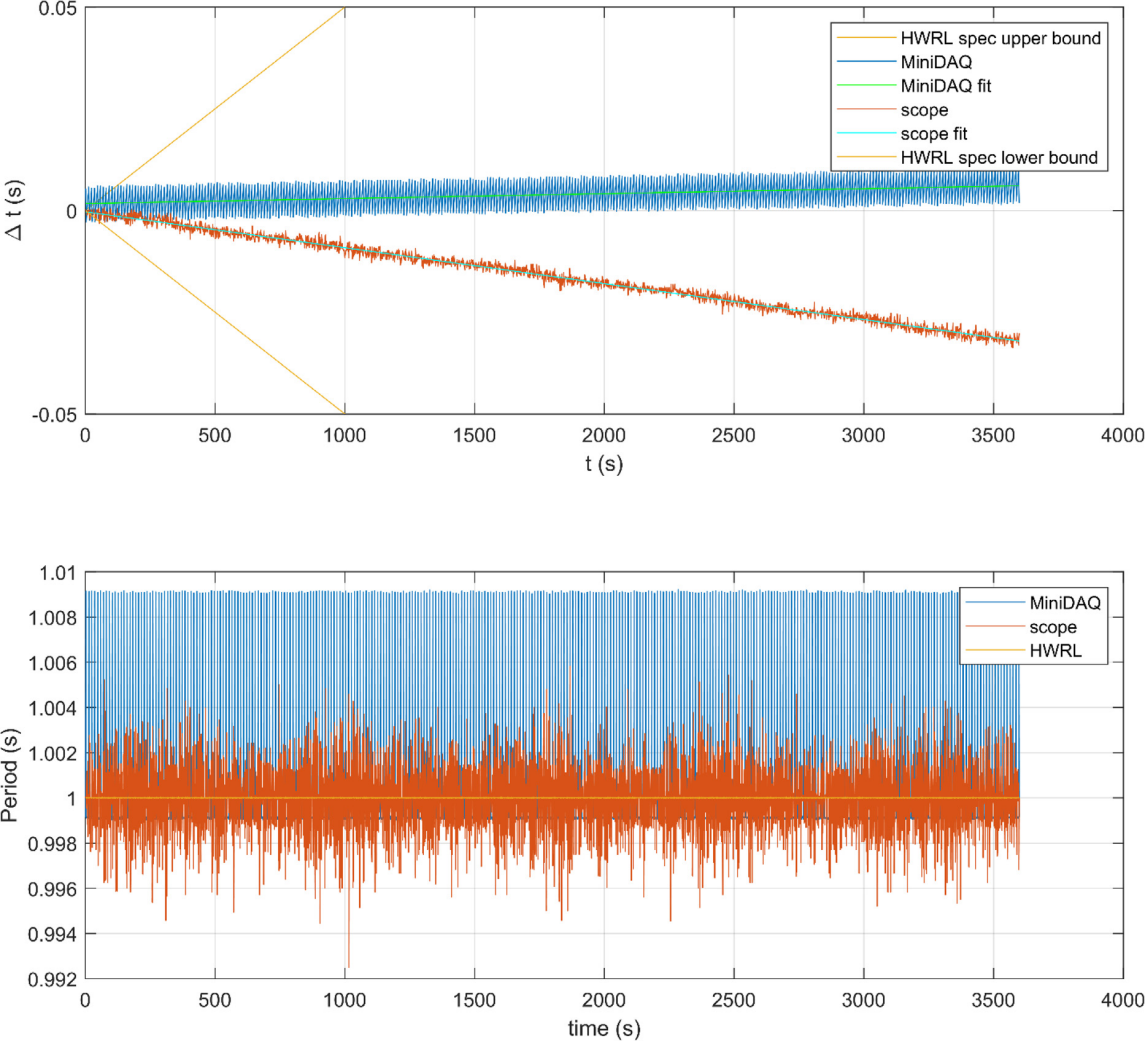
Clock drift analysis showing worst case HWRL spec and MiniDAQ and scope in relation to HWRL DAQ in top plot. Bottom plot shows individual sine wave periods vs. time.

To quantify the clock drift between the different capture methods, an analysis of the sine wave captured data was undertaken. A zero up-crossing analysis was done and the average timestamp between consecutive up-crossings was recorded for all three signals. As the HWRL data acquisition system was the most sophisticated and trusted source it was treated as the “truth” to be compared against by the other two signals. In this manner the resulting MiniDAQ and scope timestamps were subtracted from the HWRL timestamps to see how the clocks shifted over time. The top plot in Fig. 6 shows the product specification for the HWRL data acquisition clock at 50 parts per million (ppm). This is to show what would happen if at every clock cycle the clock was off exactly the specification throughout the capture duration. While this is extremely unlikely, it gives a bound to compare the other results to. A linear first order fit was applied to the scope and MiniDAQ signals to estimate a drift in ppm. Scope results show a drift of −8.83 ppm while the MiniDAQ show a drift of 1.22 ppm. These are well inside the HWRL specifications and indicate a reasonable level of clock drift.

The bottom plot in Fig. 6 shows the individual periods of the sine wave vs. time giving insight into the variations in periods over time. It is suspected that the low variation in periods achieved by the HWRL DAQ is due to the anti-aliasing filtering being done to the signal at capture. The MiniDAQ and scope do not have an equivalent filtering applied.

**Fig. 7:**
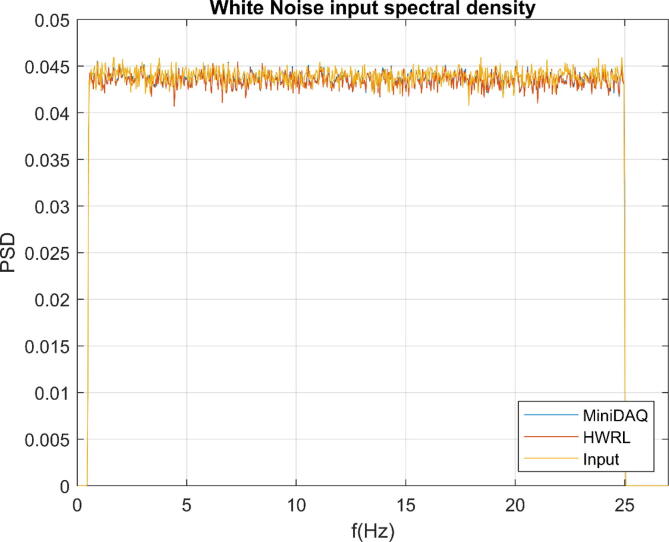
Spectral density of band limited white noise input compared between MiniDAQ, HWRL, and input signal.

Finally, a bandlimited white noise signal was created as an input for analysis. An amplitude of 5 V and a bandlimit of 0.5 to 25 Hz was applied. This signal was only captured with the MiniDAQ and the HWRL DAQ and the power spectral density (PSD) of the recorded signal is show in Fig. 7. The PSD of the input signal is included for comparison. This shows that both the MiniDAQ and HWRL DAQ can capture a signal over the frequency range chosen which corresponds to a typical range for small scale WEC research.

### CRediT authorship contribution statement

**Bret Bosma:** Methodology, Software, Data curation, Investigation, Formal analysis, Writing – original draft, Visualization. **Ryan Coe:** Conceptualization, Writing – review & editing, Supervision. **Giorgio Bacelli:** Conceptualization, Writing – review & editing, Supervision. **Ted Brekken:** Writing – review & editing, Supervision, Project administration, Funding acquisition. **Budi Gunawan:** Writing – review & editing, Project administration, Funding acquisition.

## Declaration of Competing Interest

The authors declare that they have no known competing financial interests or personal relationships that could have appeared to influence the work reported in this paper.
